# Influence of the Bracket Material on the Post-Cure Degree of Conversion of Resin-Based Orthodontic Adhesive Systems

**DOI:** 10.3390/polym16030318

**Published:** 2024-01-24

**Authors:** Ivona Profeta Krznar, Matej Par, Zrinka Tarle, Senka Meštrović

**Affiliations:** 1 School of Dental Medicine, University of Zagreb, Gunduliceva 5, 10 000 Zagreb, Croatia; profeta.ivona@gmail.com; 2 Department of Endodontics and Restorative Dentistry, School of Dental Medicine, University of Zagreb, Gunduliceva 5, 10 000 Zagreb, Croatia; tarle@sfzg.hr; 3 Department of Orthodontics, School of Dental Medicine, University of Zagreb, Gunduliceva 5, 10 000 Zagreb, Croatia; mestrovic@sfzg.hr

**Keywords:** degree of conversion, FTIR, orthodontic adhesive systems, orthodontic brackets

## Abstract

The aim of this study was to examine the influence of the orthodontic bracket material on the short-term and long-term post-cure development of the degree of conversion (DC) of resin-based orthodontic adhesive systems. Five commercially available materials characterized by different compositions and curing modes (light-curable or dual-curable) were tested under three different light curing conditions: without brackets (control group, CO), and in the presence of metal brackets (MB group) or ceramic brackets (CB group). Fourier-transform infrared spectroscopy was used to determine the post-cure DC development, both after “short-term” periods (2, 6, and 10 min) and “long-term” periods (1, 7, and 28 days). The short-term DC values ranged from 43.9% to 76.1%, and the long-term DC values were higher and ranged from 54.3% to 85.3%. The MB group demonstrated significantly lower short-term DC values compared to the CO and the CB groups, while the CB group had statistically similar or slightly lower DC values compared to the CO group. Long-term DC values in the MB and the CB groups were statistically lower or similar compared to the CO group, which depended on the post-cure time. The results indicated that the post-cure DC development was highly material-dependent and affected by the presence of different types of bracket material.

## 1. Introduction

The majority of contemporary orthodontic adhesive systems are basically resin composite materials. Among the materials used for bonding brackets, these are usually the materials of choice due to their excellent mechanical and aesthetic properties, as well as a low incidence of debonding [1]. Light-cured systems are particularly favored because of their ability to rapidly achieve high bond strength, ease of handling, and favorable aesthetic characteristics [1]. While numerous studies have examined the bond strength of orthodontic adhesive systems, there has been less emphasis on the degree of conversion (DC) [2], which measures the extent of the polymerization reaction [3]. Ideally, all monomers should be converted into polymer during the polymerization reaction, but as the movement of reactive species becomes increasingly difficult as the polymerization progresses, it is practically impossible for the DC to reach 100% [4,5].

Mechanical and aesthetic properties, such as bond strength, solubility, water sorption, chemical degradation, and color stability, are closely related to the DC [1,4,6,7,8]. A lower DC may affect the biocompatibility of the material, potentially causing allergic and toxic reactions due to the leaching of residual monomers into the oral cavity [4,9,10].

Post-cure development of the DC of resin composite materials is a well-documented phenomenon [11,12], with subsequent polymerization continuing for up to a month after the initial reaction [13]. Consequently, DC values increase over time. Previous research has shown that the DC of restorative composites increases for at least 24 h after light curing [5,10], indicating that material properties related to DC can develop over time.

Reported DC values in studies of orthodontic adhesive systems vary significantly. For instance, Çörekçi et al. reported DC values ranging from 57% to 88% [4]. Eliades et al. reported DC values ranging from 48% to 68% [2], while Üşümez et al. reported values ranging from 36% to 67% for some commercially available orthodontic adhesive systems [14].

Most of the available studies on the DC of orthodontic adhesive systems have documented the values immediately after curing [2,6,15,16,17,18,19], and studies of post-cure DC development are scarce [4]. In the study by Çörekçi et al., no significant difference was observed in DC values measured one day versus 30 days after curing for the tested materials [4]. In contrast, DC values in another study [12] were increased after one day, seven days, and thirty days compared to DC values immediately after curing for one commercial and three experimental orthodontic adhesive systems. The scarcity of studies dealing with long-term DC development and their contradictory results indicate the need for further research on this topic.

Some of the reviewed studies [4,14] have measured the DC without the presence of brackets. In clinical settings, the presence of a bracket can impede direct light passage to the material (in the case of a metal bracket), or light can be scattered (in the case of a ceramic bracket), potentially leading to variations in the material’s DC [20]. Other studies have compared differences in the DC values between metal and ceramic brackets. Reported DC values in the presence of metal brackets were found to be between 48% and 56%, which is lower when compared to ceramic brackets (ranging from 58% to 79%) [2,6,16,18], irrespective of the adhesive system used and the curing time. Shinya et al. demonstrated that the presence of metal brackets reduced DC values by 17% to 29% compared to the control group (without brackets) for two commercially available orthodontic adhesive systems [8]. However, most of these studies did not address the post-cure development of DC as a function of the bracket material and did not include a control group without an overlying bracket for comparison with the metal/ceramic bracket groups.

In addition to light-cured adhesive systems, dual-cured systems are also available on the market. In these systems, polymerization is initiated by light, while light-independent chemically initiated polymerization compensates for the lack of light exposure in the deeper layer of the material or at the sites shaded by the orthodontic bracket [7]. Consequently, these systems are expected to better tolerate insufficient light delivered to the material [9]. They have demonstrated higher bond strengths and DC values than chemically and light-cured systems [10].

While extensive research has been conducted regarding the post-cure DC development of restorative composites, few data are available regarding the post-cure DC development of orthodontic adhesive systems, depending on the bracket material used. Furthermore, most available studies lack a comparison between reference DC values achieved with no overlying material (e.g., brackets) and DC values when light curing is performed in the presence of brackets. Hence, this study aimed to evaluate the influence of the bracket material on the short-term and long-term post-cure DC development of different orthodontic adhesive systems. The null hypothesis was as follows:

The type of bracket material and the adhesive system do not influence the DC and post-cure development of the DC of orthodontic adhesive systems.

## 2. Materials and Methods

Five commercially available orthodontic adhesive systems were tested. Table 1 displays their material types and compositions.

**Figure 1:**
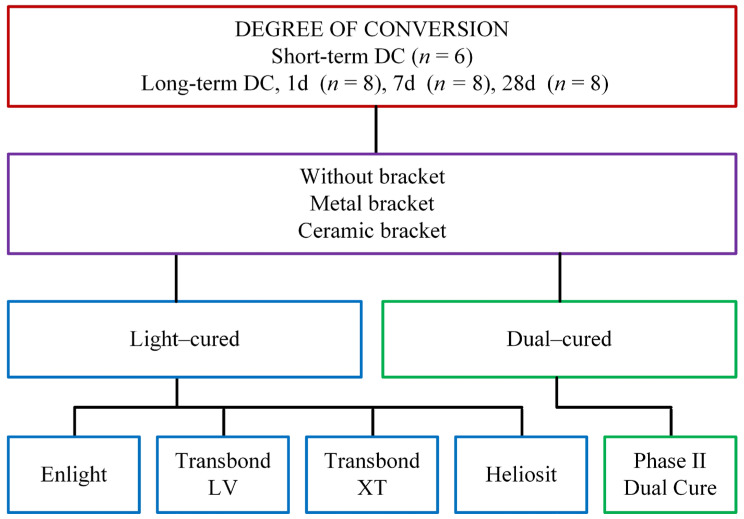
Study design.

Three experimental groups were used (Figure 1): the control group without brackets (CO), the group with metal brackets (MB; Equilibrium 2, Dentaurum Ispringen, Germany), and the group with ceramic brackets (CB; Perfect Clear II, Osstem Orthodontics Inc., Uiwang-si, Republic of Korea). Due to their flat base surface, the upper central incisor brackets were used.

To measure short-term and long-term DC development, Fourier-transform infrared spectroscopy (FTIR) with attenuated total reflectance (ATR) accessory was used, employing a Nicolet iS50 instrument (Thermo Fisher, Madison, WI, USA). FTIR spectra were collected in the 500–3500 cm^−1^ wavenumber range at a spectral resolution of 8 cm^−1^ for short-term DC and 4 cm^−1^ for long-term DC measurements. For short-term DC measurements, each spectrum was recorded in two scans, at a data collection rate of two spectra per second, with continuous spectral recording for 10 min from the activation of the light-curing unit [21]. For long-term DC measurements, one spectrum was recorded at each time point (1, 7, and 28 days), using 30 scans per spectrum.

The DC was calculated by comparing the relative change in the height of the spectral band at 1638 cm^−1^ (aliphatic C=C) with the internal standard at 1608 cm^−1^ (aromatic C=C) before and after polymerization, using the equation:

(1) $$DC\;\left(\%\right)=\left[1-\frac{\left(1638\;{cm}^{-1}\right)/{\left(1608{cm}^{-1}\right)}_{cured}}{\left(1638\;{cm}^{-1}\right)/{\left(1608{\;cm}^{-1}\right)}_{uncured}}\right]\times 100$$

The analysis of the FTIR spectra and the DC calculations were performed according to the procedure commonly referred to as “Rueggeberg’s standard baseline method” [22], which is a common method for evaluating the DC of dental resin-based materials [23,24].

### 2.1. Sample Preparation for Short-Term DC Measurements

Six samples per experimental group, i.e., for each combination of material and bracket, were tested (*n* = 6), as well as for the control group without brackets. Ninety samples in total were tested.

**Figure 2:**
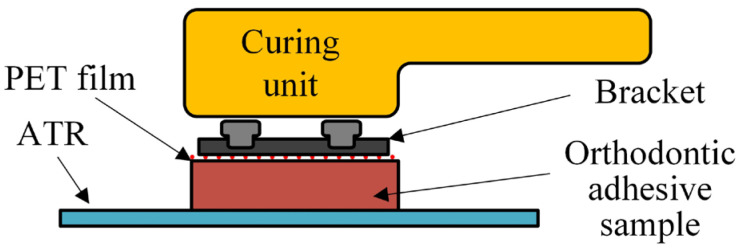
Schematic representation of sample preparation for the short-term DC measurements.

A thin layer of unpolymerized material was placed on the ATR crystal and covered with a polyethylene terephthalate (PET) film (Hawe Striproll; Kerr, Orange, CA, USA) that matched the surface of the bracket base, in order to ensure consistent pressure of the bracket on the entire surface of the material and to facilitate the removal of the bracket after completing the measurements (Figure 2). The bracket was placed on the PET film with even pressure, creating a thin layer of material of approximately 250 μm, to mimic clinical conditions [25]. Excess material was removed with a dental probe. For the samples in the CO group, the bracket was used only for thinning the material and was then removed, while the PET film was left on the sample. For the samples in the MB and CB groups, the bracket was left on the PET film. The samples were light-cured with the LED curing unit Bluephase G2 (Ivoclar Vivadent, Schaan, Liechtenstein) with a continuous intensity of 1000 mW/cm^2^ for 20 s directly above the material for the samples in the CO group, and directly above the ceramic bracket for the samples in the CB group, while samples in the MB group were light-cured for 10 s from the mesial side and 10 s from the distal side of the bracket at a 45° angle. FTIR spectra were recorded for 10 min from the start of light curing in the previously described manner.

### 2.2. Sample Preparation for Long-Term DC Measurements

Eight samples per experimental group, i.e., for each combination of material and bracket, were tested (*n* = 8), as well as for the control group without brackets. Separate samples were prepared for testing at three different time points (1, 7, and 28 days), resulting in a total of 360 samples.

**Figure 3:**
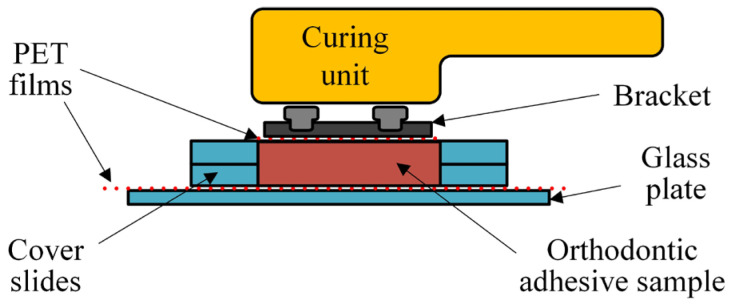
Schematic representation of sample preparation for the long-term DC measurements.

A PET film was placed on the microscope’s glass slide, onto which the material was applied. Two microscope cover slides were placed onto each side of the PET film, acting as spacers to ensure the uniform thickness of each sample. Another PET film, cut to match the surface of the bracket base, was placed on top of the material (Figure 3). The upper PET film was pressed firmly using another microscope glass slide, which was subsequently removed. The upper PET film was left on the sample in all experimental groups. The purpose of the lower PET film was to facilitate the removal and transfer of the specimen after polymerization into the black box used for storage, while the upper PET film facilitated the removal of the bracket from the surface of the specimen. In the MB and CB groups, a bracket was additionally placed on the upper PET film. Excess material was removed with a dental probe. The samples were light-cured, as previously described. After light curing, the upper PET film and the bracket (when applicable) were removed. After removing the sample from the lower PET film, it was transferred to a black plastic box and left to age under dry conditions in a laboratory incubator at 37 °C for measurements after 1, 7, and 28 days. After being stored for a particular time, the samples were withdrawn from the incubator and placed on the ATR crystal. The sample surface opposite the illuminated one was pressed onto the ATR crystal using a dedicated load-controlled press of the spectrometer. The FTIR spectra were recorded as previously described. Additionally, the spectra of unpolymerized materials (*n* = 5) were collected to calculate the DC.

### 2.3. Statistical Analysis

The normality of distribution was verified using the Shapiro–Wilk test and the inspection of normal Q–Q diagrams. Since no significant deviations from the assumption of normality were observed, a mixed-model ANOVA was used to compare the DC values with the within-subjects factor being “time point” and the between-subjects factors being “adhesive type” and “bracket type”. Due to statistically significant interactions among the factors, the DC data among the orthodontic adhesive systems and bracket types were compared using two separate one-way ANOVAs with Tukey post hoc adjustments for multiple comparisons. The comparisons among different time points were performed using repeated-measurement ANOVAs with Bonferroni post hoc adjustments. Statistical analysis was performed using SPSS 25 (IBM, Armonk, NY, USA) at an overall significance level of 0.05.

## 3. Results

**Figure 4:**
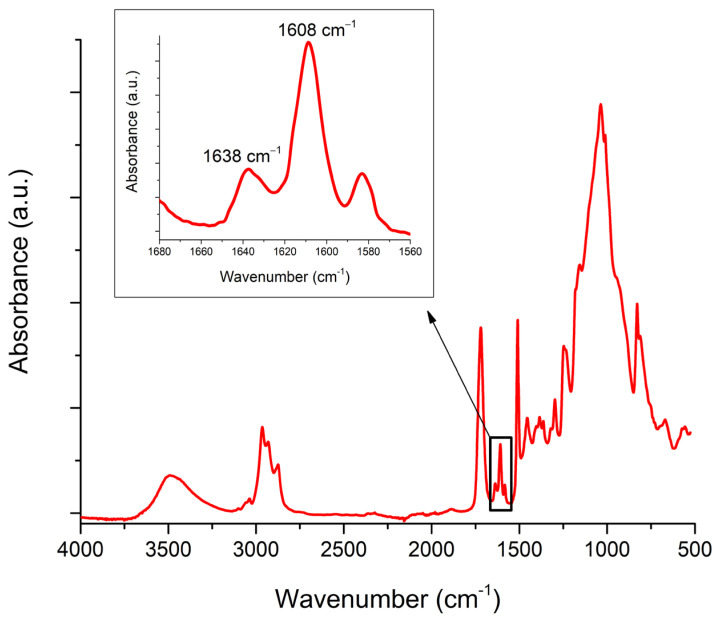
A representative FTIR spectrum of the resin-based light-cured orthodontic adhesive system Enlight. The inset shows an enlarged part of the wavelength range of interest for DC calculations, containing the vibrational modes of aliphatic C=C (1638 cm^−1^) and aromatic C=C (1608 cm^−1^).

A representative FTIR spectrum is shown in Figure 4. Despite the different FTIR spectral features of the various orthodontic adhesive systems, which occur due to their compositional differences, all materials contained spectral bands at 1638 cm^−1^ (representing aliphatic C=C) and 1608 cm^−1^ (representing aromatic C=C). The change in intensity of the aliphatic C=C band at 1638 cm^−1^ reflects the consumption of C=C double bonds in the methacrylate monomers, and its relative change compared to the initial intensity of the uncured material is used to quantify the extent of polymerization described by the DC. The aromatic C=C band at 1608 cm^−1^ is commonly used as an internal standard. This band remained unchanged throughout the polymerization.

### 3.1. Short-Term DC Measurements

**Figure 5:**
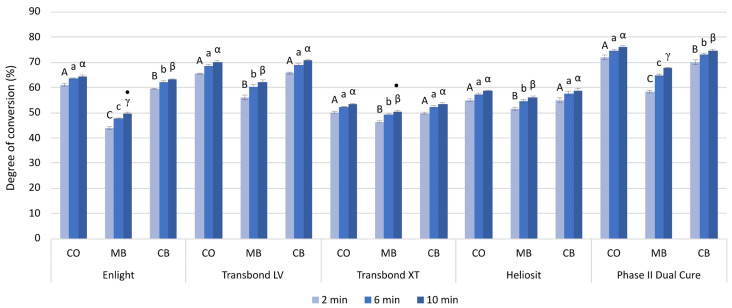
Degree of conversion (mean values and standard deviations) measured 2, 6, and 10 min after light curing for each system. For the comparisons among bracket types, the same uppercase, lowercase, and Greek letters indicate statistically similar values after 2, 6, and 10 min, respectively. Statistically similar values among the materials (within each combination of bracket type and time point) are marked by dots (·). CO: control group (no bracket); MB: metal bracket group; CB: ceramic bracket group.

The short-term DC values measured 2, 6, and 10 min post cure are shown in Figure 5.

The short-term DC values ranged from 43.9% (DC_2min_Enlight, MB) to 76.1% (DC_10min_Phase II Dual Cure, CO) and increased significantly over the 10 min observation period for all materials and all groups.

The MB group had significantly lower DC (%) values than the CO group (2.8–14.8) and the CB group (2.6–13.3). The DC values in the CB group were statistically similar to those of the CO group, except for Enlight and Phase II Dual Cure, where the CB group showed significantly lower DC (%) values (1.3–1.5).

The lowest DC values after 10 min were shown by Transbond XT in the CO group (53.4%) and CB group (53.5%), and Enlight in the MB group (49.7%). The highest DC values after 10 min were shown by Phase II Dual Cure in all three groups (CO: 76.0%, CB: 74.7%, MB: 67.8%).

The largest difference in the DC (%) values between 2 and 10 min of measurement was observed in the MB group for Phase II Dual Cure (9.4), and the smallest difference in DC (%) values was in the CO group for Transbond XT (3.2).

### 3.2. Long-Term DC Measurements

**Figure 6:**
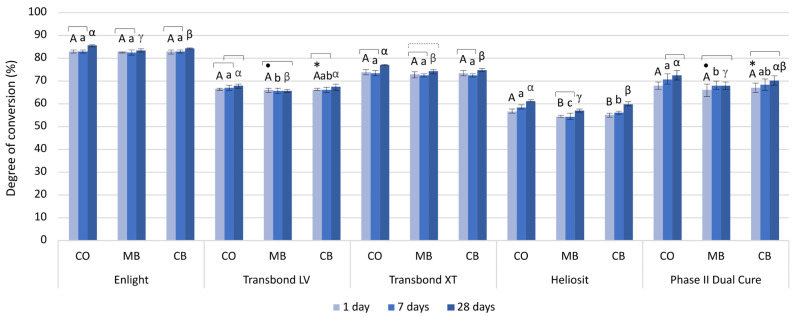
Degree of conversion (mean values and standard deviations) measured after 1, 7, and 28 days. For the comparisons among bracket types, the same uppercase, lowercase, and Greek letters indicate statistically similar values after 1, 7, and 28 days, respectively. Statistically similar values within each group are connected with square brackets. The dashed bracket indicates the values that are statistically similar between the first and the last time point, but differ from the time point in between. CO: control group (no bracket); MB: metal bracket group; CB: ceramic bracket group. Statistically similar values among the materials (within each combination of bracket type and time point) are marked by dots for the MB group (·), and asterisks for the CB group (*).

The long-term DC values measured 1, 7, and 28 days post-cure are shown in Figure 6.

The long-term DC values ranged from 54.3% (DC_1d_Heliosit, MB) to 85.3% (DC_28d_Enlight, CO).

A statistically significant increase in DC (%) values 7 days after light curing was observed for Heliosit (1.7) and Phase II Dual Cure (2.8) in the CO group, and Heliosit in the CB group (1.0). The DC (%) values in the MB group were significantly lower compared to the CO group (1.3–3.9) for Transbond LV, Heliosit, and Phase II Dual Cure.

A statistically significant increase in DC values was observed between 1 and 28 days (0.7% for Enlight, MB; 4.8% for Heliosit, CB) for most materials and groups, except for Transbond LV and Phase II Dual Cure, which showed an increase only in the CO group. The DC (%) values for Enlight, Transbond LV, and Heliosit in the MB group were significantly lower compared to the CO group (2.0–4.3) and the CB group (0.8–2.2).

The only material that showed a statistically significant difference among groups for the majority of observed measurement time points was Heliosit (except 1 day after light activation, where the MB and the CB group had statistically similar values), with the lowest values being in the MB group (DC_1–28d_ 54.3–57.0%), and the highest values being in the CO group (DC_1–28d_ 58.3–61.2%).

**Figure 7:**
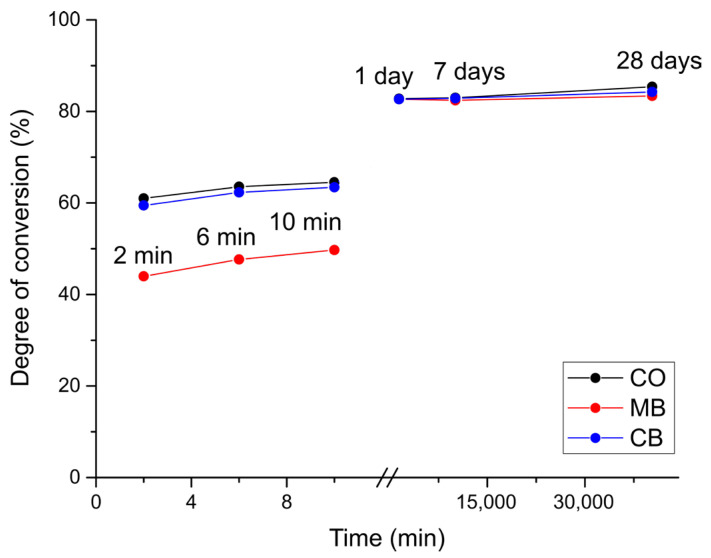
The development of the degree of conversion across both the short-term and long-term measurements (material: Enlight). Note the break in the middle of the *x*-axis.

Compared to the DC values obtained after 10 min in the short-term measurements, only Transbond XT and Enlight showed a substantial rise in DC values after 1 day (19.2–32.9%) in all groups, while Transbond LV showed a small DC (%) increase in the MB group (3.8). Heliosit showed a small increase in DC (%) after 28 days (1.0–2.7) in all groups. The DC increase over the entire measurement period (both short-term and long-term) is illustrated for the material Enlight in Figure 7.

## 4. Discussion

The retention of the bracket on the enamel surface depends on the DC of the adhesive system used for bonding [19]. This study evaluated the influence of the type of bracket material on short-term (10 min) and long-term (28 days) development of the DC of five different orthodontic resin-based adhesive systems. To evaluate the short-term DC, real-time measurements were performed during light activation and were continued for 10 min. A statistically significant effect of the type of bracket material and adhesive system on the short-term DC was identified for most of the adhesive systems and groups tested, except in the CB group, where only Enlight and Phase II Dual Cure showed statistically significant lower values compared to the CO group. To assess long-term post-cure DC development, measurements were performed 1, 7, and 28 days after light activation. A statistically significant post-cure DC increase up to 28 days following light activation was identified for most of the materials and groups tested, except for Phase II Dual Cure and Transbond LV, where only the CO group showed a statistically significant post-cure DC increase. Significantly lower DC values 28 days after light activation were found in the MB group for all materials, and Enlight, Transbond XT, and Heliosit in the CB group compared to the CO group. Hence, the null hypothesis was partially rejected for particular combinations of orthodontic adhesive systems and types of bracket material.

In this study, short-term DC values (2–10 min) ranged from 43.9% to 76.1%, while long-term DC values (1–28 days) ranged from 54.3% to 85.3%. These results indicate that DC values increase over time due to the continuous post-cure polymerization. This phenomenon occurs due to a significant increase in the viscosity of the reaction medium during the initial phase of the reaction, which reduces the subsequent polymerization rate [26]. As a result, the reaction rate drops by several orders of magnitude [27], even though there are still significant amounts of reactive species available, such as free radicals and unreacted double C=C bonds [28]. The low mobility of these species causes the post-cure polymerization to continue at a very slow and decreasing rate until the viscosity rise completely immobilizes the remaining reactive species. This leads to the polymerization stopping before all reactants are consumed [29].

The resulting ranking of materials from the lowest to highest short-term DC value in the CO and CB groups was: Transbond XT < Heliosit < Enlight < Transbond LV < Phase II Dual Cure for all time points; and in the MB group: Enlight < Transbond XT < Heliosit < Transbond LV < Phase II Dual Cure after 2 and 6 min, and Enlight ~ Transbond XT < Heliosit < Transbond LV < Phase II Dual Cure after 10 min. The ranking of materials with respect to the long-term DC was: Heliosit < Transbond LV < Phase II Dual Cure < Transbond XT < Enlight for all groups and time points tested, except after 1 day in the MB and CB group, where it was: Heliosit < Transbond LV ~ Phase II Dual Cure < Transbond XT < Enlight. Differences in the DC values between the materials tested could be attributed to variations in the materials’ compositions. Since the manufacturers do not fully disclose the exact material compositions [18], it is impossible to determine which specific ingredients contributed to a particular result [12,18]. Sample preparation and baseline determination in FTIR spectrometry are additional factors possibly affecting the DC and making comparisons among studies performed by different research groups difficult [9]. Preparing samples for the evaluation of long-term DC development involved more material manipulation, making the measurements inherently less precise compared to the short-term measurements. Also, good contact should be made between the sample’s surface and the ATR crystal in order to improve the signal-to-noise ratio. The various amounts of noise introduced by mounting the pre-cured specimens to the ATR crystal can potentially influence the DC.

It has been demonstrated that DC values impact a composite material’s mechanical characteristics as well as its resistance to chemical degradation and dissolution [30,31,32]. However, higher DC values do not necessarily mean better mechanical properties, especially when comparing materials of different compositions. Increasing the concentration of more mobile, low-viscosity “diluent” monomers can keep DC values high without improving the mechanical properties [6]. The DC value only indicates the percentage of double bonds converted into single bonds during polymerization. It does not provide comprehensive details regarding the polymer network’s structure. Generally, faster polymerization induces more polymeric chain growth centers and results in more interconnected networks with better mechanical properties [33]. On the other hand, slower polymerization leads to a more flexible and more linear polymer structure with fewer crosslinks [9]. In a study by Carek et al., the relationship of the DC and mechanical properties revealed that varying curing parameters can result in significantly different flexural strength and modulus values. Interestingly, these differences in mechanical properties were not always correlated with variations in the DC values [9]. Therefore, slight differences in DC values between materials do not necessarily indicate the performance of one material being superior to another. Comparing the DC values of a single material composition cured under different conditions has the benefit of excluding the variations that occur due to compositional differences; however, even then the relationships between the DC and macro-mechanical properties are not always correlated in a straightforward manner [9].

Among the various methods employed for DC measurement, FTIR is the most widely used technique [2,4,8,15,18,34]. This is a direct method for quantifying the DC [18,35]. It has been shown that the curing light reflection from the metal surface of the ATR accessory is lower than that from tooth enamel, hence the DC values obtained in vitro could be lower than those under clinical conditions [18]. In a preliminary study, we evaluated the reflection from the metal surface surrounding the diamond window of the ATR accessory and sound enamel at the buccal surface of a premolar tooth and found a twice-higher curing light reflection for the sound enamel. Such a result is aligned with the aforementioned work [18] and indicates that in vitro studies may indeed underestimate the clinically attainable DC values of orthodontic adhesives. However, due to the high total radiant exposure and the thin layer of orthodontic adhesive used in our study, this effect is unlikely to be of practical significance.

Several characteristics of light-curing units such as the type of light source, spectral distribution, light intensity, exposure mode, and irradiation time can affect DC values [4,12]. In the present study, samples in all groups were irradiated with the same light-curing unit, using the same light intensity and exposure time. In the CO group, the material was illuminated directly above the sample, while in the CB group it was illuminated directly above the bracket. In the MB group, the sample was illuminated from the mesial and distal sides of the bracket at a 45° angle, as it has been demonstrated that this position favors composite polymerization [19]. Such a positioning of the curing unit also corresponds to its clinical handling during the bonding of orthodontic brackets.

The lowest short-term DC value was shown by Enlight in the MB group and Transbond XT in the CO and CB groups. The DC values of these materials after 1 day demonstrated a large increase (19.2–32.9%) across all groups. According to the manufacturer, these materials have a high filler content (70–80 wt%). Because of the attenuation of light passing through the material due to light absorption and scattering on filler particles, lower radiation energy is received [25,26]. Higher filler content also limits the mobility of monomers and propagation of the polymerization reaction, resulting in a lower DC at the beginning [7,17], which consequently leads to a more extensive DC increase during the “dark” period [9]. Considering the large increase in DC values measured 1 day after polymerization for Enlight and Transbond XT in all groups, caution should be exercised when interpreting the results of other studies [6,8,10,17,18,19,36] that measured the DC immediately after light curing. Generally, DC values measured within a period shorter than 1 day should be considered as gradually developing values, which are likely to vary depending on the time interval between the light curing and performing the measurement.

Also, Enlight and Transbond XT showed the highest long-term DC values in all three groups. Higher DC values are associated with better biocompatibility [7,10,18] since a reduced quantity of potentially harmful monomers are available for leaching into the oral cavity. The ideal adhesive should reach its final DC quickly after application, as this would help to ensure that mechanical strength is achieved earlier while minimizing the risk of possible biological adverse effects [9]. The short-term DC values for Enlight and Transbond XT were the lowest in all three groups, which means that these materials could have a lower initial biocompatibility.

Phase II Dual Cure demonstrated the highest short-term DC values in all three groups. This finding is similar to the results found by Eliades et al. [2], where the tested dual-cured product (Duo Cement, Coltene) also demonstrated the highest DC. Dual-curing adhesive systems combine chemical and photochemical initiators in order to facilitate curing at sites obscured by the bracket’s opaque material and therefore not exposed to light, while maintaining the rapid on-demand setting of light-curing adhesive systems [37]. In contrast to the short-term DC values of Phase II Dual Cure, its long-term DC values in all groups ranked in the middle among the tested materials. There was no large increase in values between 1 and 28 days following light activation, which could be attributed to the dual-curing mechanism used in Phase II Dual Cure. Since two reactions occur simultaneously, more reactants may be initially consumed, leaving fewer for post-cure polymerization.

The short-term measurements indicated a statistically significant DC reduction for all materials in the presence of a metal bracket, with 2.8–14.8% lower DC values compared to the CO group. Reduced DC values due to the presence of the metal bracket was also observed in the long-term DC of all materials except Transbond XT, but the differences between groups were comparatively smaller (1.3–4.3%). The polymerization of adhesive beneath an opaque stainless-steel bracket depends on the ability of the light to penetrate the resin material and the amount of light reflected and backscattered from the background surface [8]. Under clinical conditions, the light is reflected from the tooth enamel [20,38], while in the laboratory setting, the corresponding reflection occurs from the instrument surface below the adhesive sample. Considering the small thickness of the adhesive layer (250 μm [25]), curing light reflection can considerably affect the polymerization [2]. The smaller differences in long-term DC values measured in the MB group compared to the CO group could be explained by the amount of reactants left for post-cure polymerization and the total amount of energy delivered to the material. In the presence of metal brackets, the total energy delivered to the material is lower, resulting in a smaller increase in the viscosity of the reactive medium and more reactants being available for post-cure polymerization. This is also why the long-term DC values in the MB group are similar to those in the CB group, in contrast to the short-term DC values, where all materials in the CB group had significantly higher values (2.6–13.3%). In contrast to metal brackets, ceramic brackets allow some transmission of curing light. Optimal light transmittance is the primary advantage of the monocrystalline ceramic brackets used in this study, as they lack the grain boundaries present in polycrystalline brackets, which causes light scattering and refraction [6], hence attenuating the curing light that reaches the adhesive. That is why the CB group’s short-term DC values were statistically similar to the CO group and higher than those of the MB group.

The long-term DC values for Heliosit were the lowest among all tested materials; this result was obtained in all three groups and for all measurement time points. According to the manufacturer, Heliosit has a high monomer content (85 wt%), which could be expected to contribute to high short-term DC values. Still, as more monomers are consumed at the beginning of the reaction, fewer remain for post-cure polymerization [39]. This could help explain why Heliosit shows a slight increase in DC values up to 28 days following light activation compared to short-term DC values at the end of the measurement (1.0–2.7%) and significant differences between groups 7 and 28 days after light activation.

The translucency of the orthodontic adhesive is known to affect DC values [25]. High translucency in Transbond LV and Heliosit contributes to better conversion because the refractive index between the resin and filler particles is similar [25], resulting in less light scattering. As polymerization continues, the density and the refractive index of the polymer matrix increase, approaching the refractive index of the fillers, thus reducing light scattering and increasing light transmission [25,26,40]. These are also low-viscosity materials, which generally have lower filler contents [41]. Short-term DC values for these materials were among the highest in all groups, similar to previous studies [17]. The lower viscosity allows for a better mobility of monomers and diffusion of reactive species within the material, which can enhance the polymerization process, leading to higher DC values [42], at least at the beginning of the reaction. Long-term DC values for the low-viscosity materials Transbond LV and Heliosit were not among the highest in the overall ranking of the tested materials, probably because of the aforementioned effect of monomer consumption at the beginning of the polymerization reaction.

## 5. Conclusions

Within the limitations of this in vitro study, the following can be concluded:

1. The DC values of orthodontic adhesive systems tend to increase over time due to post-cure polymerization, with a highly material-dependent extent of increase that is additionally influenced by the presence of brackets;
2. The presence of metal brackets has a more pronounced negative impact on short- and long-term DC values compared to ceramic brackets, but such an effect becomes less significant over time due to post-cure polymerization.

## Figures and Tables

**Table 1:** Compositions of the tested resin-based orthodontic adhesive systems, as provided by their manufacturers.

Material Type	Material Name	Manufacturer (LOT No.)	Abbreviation	Resin Composition	Filler Load and Composition
Light-cured resin-based orthodontic adhesive systems	Enlight	Ormco, Brea, CA, USA (9708681)	EN	Dimethacrylate monomer 20–30%	70–80% silane- treated silica
	Transbond LV	3M Unitek, Monrovia, CA, USA (9739823)	TB LV	10–15% Bis-GMA, 10–20% TEGDMA, 1–5% Bis-EMA	50–60% silane-treated ceramic, <5% silane-treated silica
	Transbond XT	3M Unitek, Monrovia, USA (9478429)	TB XT	10–20% Bis-GMA, 5–10% Bis-EMA	70–80% silane-treated quartz
	Heliosit	Ivoclar Vivadent, Schaan, Liechtenstein (Z04SHW)	HE	Bis-GMA, UDMA, decandiol dimethacrylate (85%)	14% silane-treated silica
Dual-cured resin-based orthodontic adhesive system	Phase II Dual Cure	Reliance Orthodontic Products, Itasca, IL, USA A (231267) B (224969)	PDC	Paste A: 10–30% Bis-GMA Paste B: 10–30% Bis-GMA, 5–10% TEGDMA	Paste A: 0% Paste B: 50–75% fused silica

Bis-GMA: bisphenol A digliycidylmethacrylate; Bis-EMA: bisphenol A diglycidyl methacrylate ethoxylated; TEGDMA: triethylene glycol dimethacrylate; UDMA: urethane dimethacrylate.

## Data Availability

Data are contained within the article.
